# Parental Incarceration, Attachment to Caregivers, and Young Children's Physiological Stress

**DOI:** 10.1002/dev.70076

**Published:** 2025-09-02

**Authors:** Luke Muentner, Kerrie Fanning, Kaitlyn Pritzl, Amita Kapoor, Lindsay Weymouth, Chandni Anandha Krishnan, Julie Poehlmann

**Affiliations:** ^1^ Corrections and Reentry Research Program, Justice Practice Area RTI International Research Triangle Park North Carolina USA; ^2^ University of Wisconsin–Madison Madison Wisconsin USA

**Keywords:** attachment, cortisol, cortisone, jail, parental incarceration

## Abstract

Parental incarceration can be stressful for children, leading to less optimal development. Although parental incarceration typically takes place among other adversities, resilience processes occur in many families, for example, in instances of secure child–caregiver attachment relationships. Yet, it is not known how secure attachments are associated with stress processes in children with incarcerated parents, especially in the context of other risks. The current study analyzed data from 67 child–caregiver dyads, measuring cumulative stress hormones (cortisol and cortisone) through children's hair samples and assessing attachment security using the Attachment Q‐sort. Results indicated that children with higher attachment security scores had lower levels of cortisone and the combined cortisol/cortisone variable, aligning with the Learning Theory of Attachment, which posits that secure attachments mitigate stress through consistent, supportive caregiving. Conversely, children with insecure attachments exhibited more extreme cortisone levels, suggesting dysregulated stress responses. These findings underscore the importance of attachment security as a resilience factor in children facing parental incarceration and other adversities. The study calls for targeted interventions to strengthen caregiver–child relationships, which could buffer the long‐term negative impacts of chronic stress. These results highlight the need for family‐related approaches to support the well‐being of children affected by mass incarceration.

## 1 Introduction

Early exposure to toxic stress can have profound effects on young children's development (Danese and McEwen 2012; McEwen 2012), necessitating studies of stress processes in young children who have experienced adversity (Shonkoff et al. 2012; Szilagyi and Halfon 2015). Children whose parents have spent time in prison or jail are exposed to more adverse childhood experiences (ACEs) than other children (Turney 2018), making them potentially more vulnerable to toxic stress. Although there is much speculation about the role of stress processes in children and families affected by parental incarceration (Arditti 2016; Turney 2014), only a few studies to date have assessed physiological stress in incarcerated parents (e.g., McClure et al. 2015) or their children (i.e., Muentner et al. 2021). To our knowledge, no studies have looked at associations between children's physiological stress and potential protective factors in children with incarcerated parents, despite previous research discussing how children's experiences with adverse life events can act as mechanisms behind their attachment processes in the wake of parental incarceration (Murray and Murray 2010). To further explore possible risk and resilience factors within this context, the current study assesses how young children's physiological stress during parental incarceration is associated with experiences of adversity and their attachment to at‐home caregivers (henceforth referred to as “caregivers”), exploring the potential protective effect of attachment security.

### 1.1 Parental Incarceration in the United States

The number of people incarcerated in prisons and jails in the United States has increased dramatically over the last 40 years, with most incarcerated individuals being parents (Maruschak et al. 2021; Shlafer et al. 2019). Because of stark economic and racial disparities in mass incarceration, children of color and children living in low‐income households are much more likely to experience parental incarceration than White or economically advantaged children (Muentner et al. 2023). With more than 8 million admissions annually, and more than 10 million annual admissions prior to the COVID‐19 pandemic (Minton and Zeng 2021), a jail stay is the most common type of incarceration in the United States. Because jails are intended for short‐term or temporary custody placements that are typically less than 1 year, though this can vary depending on sentencing, individuals incarcerated in jail often experience multiple brief stays over time. In contrast to the longer‐term nature of prison stays, this pattern can contribute to greater disruptions in family life. Even when jail stays are short, the incarceration of a parent in jail can be particularly disruptive for young children's sense of security and stability of family relationships (Poehlmann‐Tynan and Arditti 2018). Longitudinal studies show that it is particularly challenging for children's development and well‐being in adolescence and young adulthood when parental incarceration occurs during a child's early years, although mechanisms of such effects are understudied (Poehlmann‐Tynan and Turney 2021).

### 1.2 Risks and Physiological Stress

The incarceration of a parent in childhood is linked with numerous adverse outcomes for children's well‐being (Poehlmann‐Tynan and Turney 2021). Compared to their peers, children with incarcerated parents exhibit comparatively high externalizing (e.g., aggression) and internalizing (e.g., depression, anxiety) behaviors, in addition to numerous health conditions, academic difficulties, and high‐risk behaviors continuing into adolescence and adulthood (Wakefield and Wildeman 2013). Families affected by parental incarceration often experience additional adversities, including financial hardship, residential instability, stress associated with parenting, and caregiver health and mental health concerns, although some of these adversities may predate the incarceration (Haskins et al. 2018; Turney and Haskins 2019; Poehlmann‐Tynan et al. 2017). In an analysis of data from a nationally representative sample of children aged 0–17 years, Turney (2018) found that children with incarcerated parents were exposed to nearly five times more ACEs, on average, than their peers, with the strongest association between parental incarceration and ACEs occurring for children in the 0–6 year range.

Scholars have hypothesized that individual and family stress processes may underlie the negative impacts of parental incarceration on children (Arditti 2016; Turney 2014). This is because development of the physiological stress system in early childhood sets a foundation for how children respond to stressful events in the future (Hostinar and Gunnar 2013). These stress processes may help explain long‐term impacts of a parent's incarceration during early childhood, which in many cases appear to magnify over time (Arditti 2016; Turney 2014; Poehlmann‐Tynan and Turney 2021). However, there are currently few published studies including physiological stress measures in children with incarcerated parents, and thus, these hypotheses remain largely untested. Our preliminary work with young children with incarcerated fathers suggests that children who witnessed their fathers’ arrest exhibit higher levels of cumulative stress hormones compared to children who did not witness the arrest (Muentner et al. 2021), pointing to differential impacts of parental incarceration on children's physiological stress depending on individual experiences, including additional adversity as well as protective factors.

#### 1.2.1 Physiological Stress

Physiological stress responses can be assessed by measuring cortisol and cortisone, glucocorticoid hormones that reflect the body's hypothalamic–pituitary–adrenal (HPA) axis activity, in the saliva, blood, urine, or scalp hair (Iqbal et al. 2023; Xie et al. 2011), with correspondence between measurement types (e.g., Kapoor et al. 2018). As the body responds to stressors in both children and adults, acute cortisol and cortisone reactions are normal and adaptive. However, chronic stress can cause longer‐term changes in hormone secretion that can negatively affect children's developing brains and stress systems in ways that lead to structural and functional changes, especially during early childhood (Smith and Pollak 2020). Longer‐term negative changes can include hyper‐ and hypoactivation of the HPA axis, consisting of chronically high levels of cortisol/cortisone or a pattern of very low or undetectable levels of cortisol/cortisone, respectively. The latter pattern, often referred to as a “blunted” stress response, reflects dysfunction in stress regulation in that bodily systems no longer respond to stressors with a normative temporary rise in stress hormones (Phillips et al. 2013). Blunted stress responses have been documented in prior studies of people experiencing posttraumatic stress disorder (PTSD) (e.g., Metz et al. 2020) and are associated with increased ACE exposure (e.g., Voellmin et al. 2015).

Numerous studies have reported hair cortisol and cortisone findings in children and adults (e.g., Bates et al. 2017; Dettenborn et al. 2012; Groeneveld et al. 2013). For instance, there is evidence of elevated hair cortisol levels among NICU infants put on mechanical ventilation, elementary school girls who experienced adversity, and individuals exposed to chronic stress (Stalder et al. 2017; Vanaelst et al. 2013; Yamada et al. 2007). A review by Vives and colleagues (2015) added that fluctuations in stress levels could be assessed through changes in hair cortisol, and a recent study showed a significant decrease in hair cortisol in infants and young children whose parents participated in a stress‐reduction intervention (Poehlmann‐Tynan et al. 2020). Of note, in our investigation of children with incarcerated fathers (Muentner et al. 2021), children with existing behavioral stress symptoms (prior to their father's incarceration) who also witnessed his arrest and were visibly distressed about it had remarkably low or even undetectable levels of cumulative stress hormones, consistent with a blunted stress response. This stress response is similar to the blunted stress response observed in children who have experienced significant trauma or adversities such as maltreatment (Gunnar 2021).

There are a number of reasons to evaluate both hair cortisol and cortisone. Studies that have measured both cortisol and cortisone have demonstrated associations with outcome measures with either one or both of the corticosteroids (e.g., Staufenbiel et al. 2015; Vehmeijer et al. 2021; Voegel et al. 2021). Some have suggested that hair *cortisone* may be a better marker of circulating cortisol as saliva cortisone is more highly associated with serum‐free, unbound cortisol (Feeney et al. 2020; Perogamvros et al. 2010). This may be due to the relative contribution of hair cortisone, as separate studies to determine the biological validity of hair cortisol have shown that circulating cortisol is incorporated into the hair as both cortisol and cortisone (Kapoor et al. 2018; Keckeis et al. 2012). Furthermore, 11ß‐HSD2—which converts cortisol to cortisone—is found in the hair follicle, suggesting that there may be direct conversion to cortisone at the time that the glucocorticoid is incorporated into the hair (Ito et al. 2005). Thus, including both measures offers a more nuanced look into the avenues by which adversity may get under the skin of young children, noting the ways in which certain protective factors may curb some of this risk.

### 1.3 Promotive and Protective Processes, Stress, and Attachment

Despite experiencing multiple stressors, many children and families with an incarcerated parent show resilience (Arditti and Johnson 2022), or the process of achieving well‐being and growth despite the presence of significant adversity or risk (Masten 2018). Research has documented the importance of the parenting system impacting children's resilience, such as secure attachment relationships with primary caregivers (see Masten [2018] for a full review). Research has started tapping into the potential for secure attachment relationships to buffer the impacts of adversity (e.g., Gunnar 2017); however, more work is needed. Indeed, Tyrell and Masten (2022) emphasize the need for additional research that explores and promotes positive parent–child attachment bonds, particularly in the context of structural inequality. Johnson and colleagues (2018) examined attachment and salivary cortisol in 177 toddlers during well‐child checkups with inoculations. For toddlers living in poverty, those who had secure attachments to caregivers had significantly lower cortisol levels than the toddlers living in poverty with insecure attachments, even when accounting for negative life events. While attachment security often serves as a promotive factor (i.e., a mechanism that supports the well‐being of children, regardless of risk experiences), child–caregiver attachment security may be an especially salient protective factor for mitigating adverse impacts to children's physiological stress within the context of parental incarceration and other negative life events. However, this has yet to be explored among this often hard‐to‐reach population.

When experiencing the incarceration of a parent, research has found that children's interactions with their caregivers are important for their well‐being and developmental outcomes (e.g., Dallaire et al. 2012; Poehlmann 2005; Shlafer and Poehlmann 2010). Yet, despite this acknowledgment of the importance of children's attachment relationships before, during, and after parental incarceration (Murray and Murray 2010; Poehlmann‐Tynan and Arditti 2018), few studies have assessed attachment in children with incarcerated parents. The few exceptions (e.g., Dallaire et al. 2012; Poehlmann 2005; Poehlmann‐Tynan et al. 2017) have found that, while rates of attachment insecurity for children with incarcerated parents mirror those of other studies in contexts of high risk (e.g., poverty, maternal depression), attachment interventions can help promote well‐being for children with incarcerated parents (Byrne et al. 2010). This suggests that children's attachment relationships may be an effective change mechanism for supporting children's well‐being, as documented in other high‐risk contexts (e.g., Bernard et al. 2015). Other work discusses the important interplay between children's socioemotional health, caregiver responsivity, and family relationships in the context of parental incarceration (Poehlmann et al. 2008). However, we do not yet know if children's attachments to their caregivers are associated with children's physiological stress responses during parental incarceration, especially under the conditions of high adversity exposure.

The Learning Theory of Attachment (LTA; Bosmans et al. 2020) was developed to integrate what we know about attachment constructs, like parental sensitivity and children's safe‐haven and secure‐base behaviors, with learning theory and neuroscience to explain how children develop attachments to their caregivers at a more micro level. LTA frames parent–child interactions following a child's experience of distress as “learning events.” During such learning events, the child's stress is lessened when the parent or caregiver sensitively responds with support. The child's feelings of fear and distress are assuaged, and the child experiences relief and comfort instead (Sroufe and Waters 1977; McQuaid et al. 2016). According to the LTA, changes in neuroendocrine activity, such as lowered cortisol/cortisone and increased oxytocin (Hostinar and Gunnar 2013; Feldman and Bakermans‐Kranenburg 2017), accompany such feelings of comfort after learning events. Over time, repeated learning events lead the child to feel increasing trust in the caregiver and to expect the caregiver to respond in a supportive manner, consistent with secure attachment or a secure‐base script. Thus, one would expect children who are securely attached to their caregivers to show lower levels of cumulative stress hormones.

### 1.4 Current Study

In the present study, we examine the associations between children's experiences of adversity, attachment security, and stress hormones, with the goal of examining attachment security as a resilience factor for young children experiencing parental incarceration. Our research questions consider: (1) Among this sample of young children with incarcerated parents, what other adverse experiences have they been exposed to? (2) What is the association between cumulative adverse childhood events, attachment, and children's stress hormones? For Research Question 2, we plan to test whether these associations are linear (e.g., more adversity and insecure attachments being associated with higher cortisol and cortisone) or quadratic (e.g., high levels of adversity and insecure attachment being associated with both [a] very high cortisol and cortisone and [b] very low cortisol and cortisone, with the latter signaling a blunting effect) (Gunnar 2021; McLaughlin et al. 2016). Based on previous work using these data, in which a blunting effect was found (e.g., Muentner et al. 2021), we hypothesize that the quadratic association will be significant, in which case we will use the quadratic term in subsequent analyses. And, finally, (3) Does attachment security have a main effect on stress hormones, or does it interact with adversity in predicting stress hormones? The third hypothesis pertains to the promotive and potentially protective nature of attachment security for children experiencing adversity, similar to findings in previous research (e.g., Johnson et al. 2018). From a statistical standpoint, a significant main effect of attachment on children's stress hormones in the expected direction would be reflective of a promotive effect of attachment security. This would suggest that, in general, attachment security is associated with lower levels of stress hormones. Alternatively, a protective effect of attachment security would be reflected by an interaction effect of attachment security and adversity on stress hormones; to be considered protective, resilience processes would be associated with positive well‐being when more adversity is present (above and beyond a promotive effect). Thus, we hypothesize that there will be a significant main effect of attachment security on children's cortisol and cortisone levels, suggesting a promotive effect. In addition, we hypothesize there may be an interaction effect between attachment security and adversity on stress hormones, wherein attachment may be protective for children experiencing the highest adversity.

## 2 Methods

### 2.1 Data and Procedures

The current study included incarcerated parents who were housed in three Midwest county jails (one was urban, one was rural, and one held a mix of individuals from both locales), all of which were overseen by each county's sheriff's department. Jail administrative staff helped the research team identify parents who had children between the ages of 2 and 6 years. Interested parents met with research staff in a private room in the jail to determine eligibility, including (1) being 18 years or older; (2) having a child in the same or adjacent county; (3) retaining parental rights; (4) having not committed a crime against the child; (5) playing a parenting role before coming to jail; and (6) speaking and reading English. If interested parents met these criteria, they were invited to participate. Following a formal consent process, a thorough interview was conducted with parents within a private area of their housing unit; due to jail protocol, jailed parents were unable to be compensated for their participation. Parents also signed release forms to contact the child's caregiver and provided written permission for the child's participation. If parents had more than one child in the age range, one was chosen at random. Those caregivers who consented and children who assented were assessed at home by two trained researchers in visits that lasted, on average, 2–3 h, although some lasted longer or were scheduled across 2 days based on family needs. One researcher interviewed the caregiver, while the other assessed the child. Caregivers were paid $50 following the home visit, and children were given an age‐appropriate book. The study was approved by the Institutional Review Board of the University of Wisconsin–Madison (SE‐2010‐0812), and a National Institutes of Health Certificate of Confidentiality was used.

### 2.2 Participants

The current study sample is drawn from a larger study involving 165 incarcerated parents, along with 86 caregivers and 86 children (e.g., Muentner et al. 2019; Poehlmann‐Tynan et al. 2017). Of these, only 67 caregiver–child dyads participated after IRB approval was obtained for collecting children's physiological stress data. The current analyses focus on these 67 dyads. Although only 49 (73%) of these children had hair cortisol and cortisone samples, we used a multiple imputation procedure to estimate missing data. Reasons for missingness include children declining assent to hair collection, having shaved heads, or hair samples being too small. Of the subsample of 67 dyads, the majority of parents in jail were fathers (88.1%) and 11.9% were mothers. Jailed parents ranged from 21 to 46 years old (*M* = 30.36, *SD* = 6.63), and 50.7% identified as Black, 25.4% as White, 3.0% as Latinx, and 16.4% as other. A little less than a fifth of jailed parents (19.4%) did not hold a high school diploma, a third graduated from high school or received an equivalency diploma (34.3%), and 41.8% completed some college or received a college degree. The majority of jailed parents (76.1%) lived with the child prior to their incarceration. Over half of jailed parents (59.7%) had experienced additional separations from the target child prior to the current incarceration.

Caregivers ranged in age from 20 to 62 years old (*M* = 31.46, *SD* = 9.41), and the majority of caregivers were the child's mother (80.6%), while 11.9% were grandmothers, 4.5% fathers, 1.5% aunts and uncles, and 1.5% other. Most caregivers identified as White (46.3%) or Black (38.8%), 6.0% identified as Latinx, and 9.0% as other. Nearly 20% of caregivers completed partial high school, 31.3% completed high school or received an equivalency diploma, 44.8% completed some college, and 4.5% graduated from college. Caregivers’ annual income ranged from $0 to $140,000 (*M* = $15,305.16, *SD* = $20,180.16). Children ranged from 2 to 6 years old (*M* = 4.02, *SD* = 1.32), and 50.7% were identified as male and 49.3% as female. Most of the children were identified as Black (40.3%) or White (25.4%), while 11.9% of children were identified as Latinx and 22.4% as another race.

About two thirds of children (68.7%) had not experienced a separation from their current caregiver. Of the children who did experience a separation from their caregiver (*n* = 21), the number of separations, age at which the separation occurred, and the length of separation varied. Eleven children experienced only one separation from their caregiver, and their age at the time of separation ranged from less than a month old to 4 years old. The remaining five children experienced between two and three separations, generally between the ages of newborn to 6 years old. Two caregivers reported they were unsure how old the child was at the time of separation. Overall, the length of separations was generally short, lasting between a few days to 3 months. A couple of separations (*n* = 6) lasted for 5 months up to 3 years. During these separations, children in general lived with their other parent or extended family, such as grandparents or aunts and uncles, though at least one child had been placed with a foster family.

### 2.3 Measures

#### 2.3.1 Negative Life Events

We asked caregivers to complete the Life Experiences Questionnaire (LEQ; Masten et al. unpublished manuscript; Masten et al. 1993) about focal children's experiences of adversity in the past 12 months. Caregivers responded in a “yes” or “no” fashion. The 31‐item LEQ included traditional ACE questions (i.e., parental divorce, substance abuse, and mental illness) and questions about homelessness, eviction, and government funds being cut off. Because some items involved situations that required adaptation but were not inherently negative for the child (e.g., mother started working, birth of a sibling), similar to Cutuli et al. (2017), we extracted the negative events that map more closely onto ACE measures. The resulting 24‐item index had a Cronbach's alpha of 0.68.

#### 2.3.2 Children's Attachment to Their Caregivers

The Attachment Q‐sort version 3.0 (AQS; Waters 1995) is an attachment measure that is based on observations of young children naturally interacting with their caregivers in the home. It focuses on a range of attachment‐related behaviors, including secure‐base behaviors, exploration, emotional responses, and social cognitions. Meta‐analyses have indicated that the Q‐sort, when used by trained observers, is reliably associated with children's Strange Situation security classifications and with parental sensitivity (Van Ijzendoorn et al. 2004). To minimize response bias, the 90 items are sorted into a fixed distribution (10 piles of nine items each) based on the salience of children's attachment behaviors, relative to other behaviors (Waters and Deane 1985). Each child's security score is calculated as the correlation between the child's Q‐profile and the Criterion Security Q‐sort (a composite of attachment expert ratings). The score ranges from +1.0 (very secure) to −1.0 (very insecure) regarding their relationship with the attachment figure assessed. Because no natural cutoff score exists for the Q‐sort for the age range of children studied, we used the continuous security scores.

All Q‐sorts were completed by a trained researcher during home visits. Ten cases were independently rated by two trained researchers, resulting in an intraclass correlation coefficient (ICC) of 0.72, which indicates good interrater reliability. The home visit included an interview with the child's caregiver, an assessment of the child's cognitive skills and vocabulary, and a 15‐min free‐play period during which the dyad was asked to play with a standard set of toys provided by the researcher. The caregiver and child were instructed to act naturally throughout the course of the visit, and the trained researcher occasionally recorded observations of Q‐sort behaviors throughout the visit. Following the home visit, the researcher immediately completed the Q‐sort for the focal child.

#### 2.3.3 Children's Physiological Stress Responses

We measure cumulative stress hormones in children's scalp hair, a noninvasive method of collecting data that results in evidence reflecting months of neuroendocrine activity rather than short‐term activity measured in saliva and other bodily fluids (Raul et al. 2004; Russell et al. 2012; Staufenbiel et al. 2013). After completing all observations and assessments with the child at the home visit, researchers used stainless steel scissors to cut hair from four sampling areas on the child's scalp within their posterior vertex (where there is the least variability in hair growth rate; Wennig 2000). The hair was measured, and up to 3 cm most proximal to the scalp was stored in aluminum foil at room temperature for analysis. The scissors were wiped with ethanol swabs before and immediately following each sample to diminish cross‐contamination (Vaghri et al. 2013).

Although scalp hair growth rates vary across people, about 1 cm of hair growth proximal to the scalp is often used to indicate the mean of 1 month's growth (Stalder and Kirschbaum 2012). Therefore, measuring glucocorticoids in a 3‐cm hair sample typically reflects cumulative HPA axis function and stress reactivity across several months (Stalder et al. 2012). Positive correlations between hair cortisol concentration and salivary cortisol have been validated in a number of studies with humans as a method of physiological stress measurement (e.g., Xie et al. 2011; D'Anna‐Hernandez et al. 2011). Analyses were conducted after winsorizing the cortisol and cortisone variables to the 1st and the 99th percentiles to correct for outlier data. In our analyses, we use three physiological stress variables: (1) hair cortisol concentration (pg/mg), (2) hair cortisone concentration (pg/mg), and (3) the standardized sum of cortisol and cortisone concentrations.

Sample hair weights ranged from 3.8 to 110.4 mg. Except for the smallest hair sample (3.8 mg), all other hair samples weighed over 5 mg (the smallest weight in which hormones are detectable in hair using mass spectrometry) and produced valid estimates of systemic cortisol and cortisone. Often, undetectable values are the result of hair sample weights below 5 mg; although most samples weighed over this threshold, seven cortisol values and two cortisone values of the hair samples contained hormone levels that were too low to be analyzed within a range of detection. In cases such as these, when undetectable values are present and hair sample weights exceed 5 mg, standard procedure in mass spectrometry is to use the lowest detectable hormone value for each individual hair sample based on weight.

Hair samples were taken to the University of Wisconsin National Primate Research Center for analysis using a liquid chromatography–tandem mass spectrometry approach (Kapoor et al. 2014, 2016). Hair samples were placed into tubes, washed twice with 2‐propanol, dried, and then ground into a powder. The hair was precisely weighed and placed into a glass culture tube and stored in the dark at room temperature until extraction. For the extraction of steroid hormones from hair, methanol and an internal standard were added to the tube of ground hair and incubated overnight. Following incubation, tubes were vortexed and centrifuged, and the supernatant was removed and run through solid‐phase extraction followed by liquid‐phase extraction. The organic phase was placed in a clean test tube, evaporated to dryness, and then resuspended in the mobile phase. All samples were analyzed using a QTRAP 5500 quadrupole linear ion trap mass spectrometer (Sciex). Chromatographic separation was performed using a Kinetex C18 column. All data were processed with Analyst software. Intra‐ and interassay coefficients of variation for this method are 4.3 and 9.2 for cortisol and 3.7 and 11.3 for cortisone, respectively.

### 2.4 Plan of Analysis

To address missingness, we implemented a multiple imputation procedure (Raghunathan et al. 2001; Van Buuren 2007), generating 10 datasets in which missing values were randomly produced conditional upon other variables in the analysis. Subsequent analyses were applied to all 10 datasets, with pooled results, based on the application of Rubin's (1987) rules, reported. (Findings were similar for the original analyses ignoring missingness and the pooled analyses.)

We begin with descriptive analyses of adversity, then move to an examination of the bivariate associations between attachment security and stress hormones using scatterplots and curvilinear analysis. We conclude by performing an OLS multiple regression analysis to address the study hypotheses.

## 3 Results

### 3.1 Adverse Experiences

Caregiver‐reported adverse life events for children ranged from 0 to 12, with a mean of 5.16 and a standard deviation of 2.89 (see Table 1). In addition to experiencing the ACE of parental incarceration, 46.3% of children experienced 0–3 additional adverse events, and 53.7% of children experienced four or more additional adverse events. Most frequently reported additional adverse events included family financial difficulty (71.6%), parental substance use challenges (41.8%), and parental job loss (44.8%). The least frequently reported events included a sibling becoming seriously ill or injured (4.5%) and a close friend of the child passing away (3.0%), and no families reported the death of a parent or sibling, a family member running away from home, or a sibling initiating the use of a substance.

**TABLE 1:** Caregiver‐reported negative life events for children in the past year.

Negative or uncontrollable life event in the past year	Count	%
A parent or sibling became seriously ill or was injured	9	13.4
Child was the victim of violence	4	6.0
A family member was the victim of violence	8	11.9
Child's parent died	0	0
Child's sibling died	0	0
Child's grandparent died	6	9.0
Child's close friend died	2	3.0
Family member ran away from home	0	0
Parents separated or divorced	19	28.4
Parent had problems at work or lost their job	33	49.3
Family financial situation was difficult	48	71.6
Family had funds stopped by a government agency	23	34.3
Family was evicted from their home or apartment	8	11.9
There were many arguments among adults living in the home	25	37.3
There were many arguments between a parent and a former/separated spouse	22	32.8
A family member attempted suicide	4	6.0
A family member developed severe emotional problems	15	22.4
A sibling became involved with drugs or alcohol	1	1.5
A parent had trouble with alcohol or drugs	28	41.8
A sibling was arrested or went to jail	6	9.0

*Note:* Negative or uncontrollable life event items used from the Life Experiences Questionnaire (LEQ) included in the analyses. Some items have been combined or reworded for clarity.

### 3.2 Stress, Adverse Experiences, and Attachment

**FIGURE 1:**
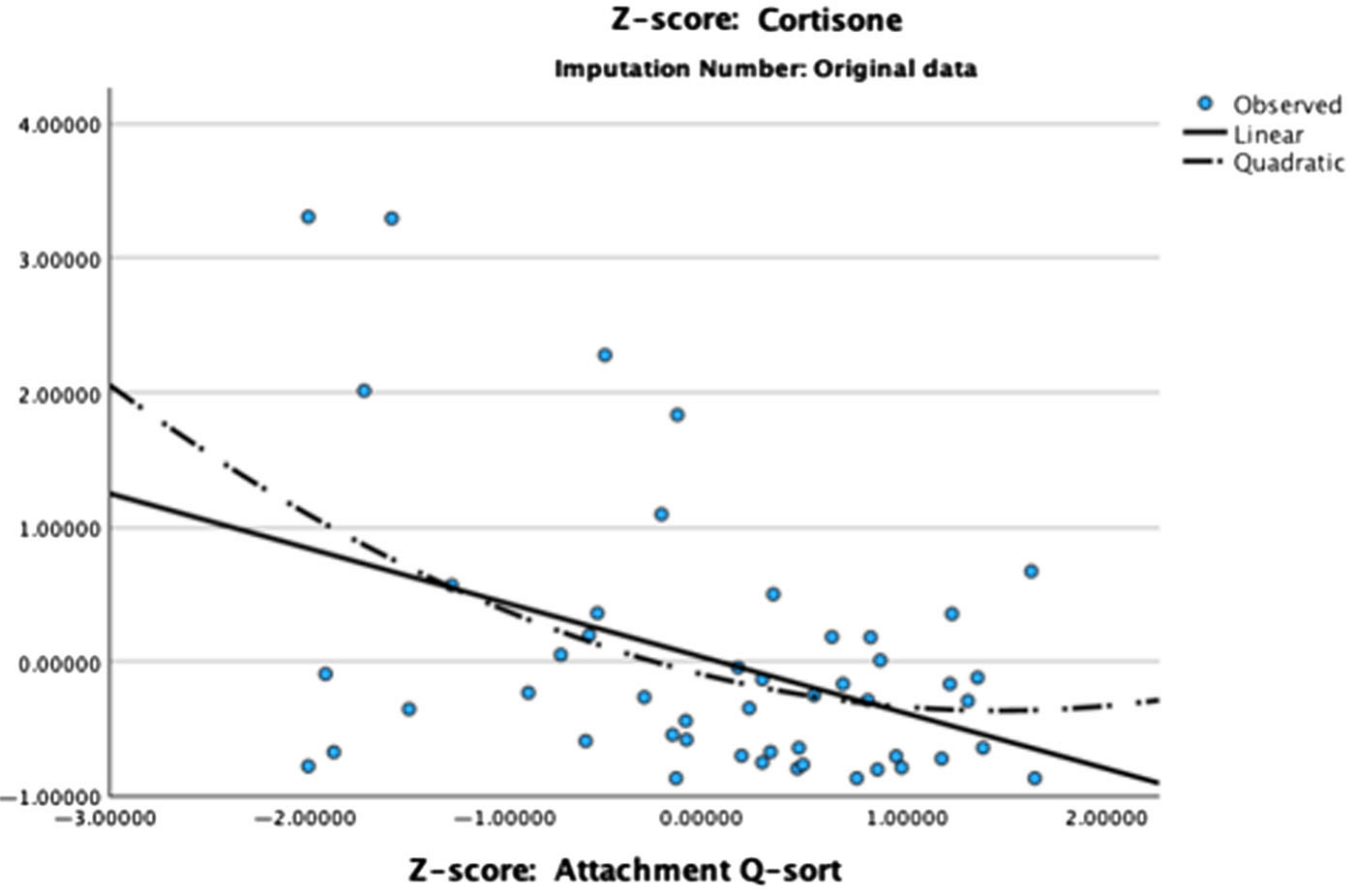
Scatterplot of the association between Attachment Q‐sort scores and cortisone (standardized variables).

Scatterplots of the AQS and adversity scores in the context of cumulative stress hormones were examined. In addition, a curve estimation procedure was used to determine if the variables were associated in a quadratic manner. For the adversity score, there was no significant linear or quadratic association with cortisol or cortisone. For the AQS score, there was a significant linear association with cortisone, *p* = 0.003, but not cortisol, as well as a significant quadratic association with cortisone, *p* = 0.008, but not cortisol (see Figure 1 for a scatterplot of the association between AQS scores and cortisone).

The linear association indicated that children with higher security scores on the AQS had lower levels of cortisone overall. The quadratic association indicated that children with the lowest security scores showed the most extreme levels of cortisone—that is, children with insecure attachments showed both the lowest and highest levels of cortisone.

### 3.3 Attachment as a Predictor of Stress

Three multiple OLS linear regression analyses were conducted to test the main and interactive associations of attachment and adversity in the prediction of cumulative hair cortisol, cumulative hair cortisone, and the sum of standardized hair cortisol and cortisone. In the first step, the predictors were the adversity score, the AQS variable, the AQS quadratic term, the interaction between the AQS and adversity score, and the interaction between the AQS quadratic term and adversity score. In a second step, children's age was added as a covariate.

In the cortisol analysis, none of the variables were statistically significant (see Table 2).

**TABLE 2:** Regression results for hair cortisol (pooled, *N* = 67).

Variable	*B*	*SE*	*β*	*t*	*p*
Model 1					
Intercept	0.055	0.127		0.436	0.664
LEQ	−0.113	0.260	−0.148	−0.434	0.666
AQS	−0.117	0.193	−0.150	−0.607	0.544
AQS^2^	−0.076	0.176	−0.057	−0.432	0.666
LEQ × AQS	0.056	0.160	0.061	0.351	0.726
LEQ × AQS^2^	−0.065	0.261	−0.054	−0.248	0.805
Model 2					
Intercept	0.052	0.127		0.409	0.683
LEQ	−0.103	0.261	−0.146	−0.395	0.694
AQS	−0.092	0.201	−0.095	−0.459	0.646
AQS^2^	−0.073	0.177	−0.033	−0.414	0.679
LEQ × AQS	0.051	0.161	0.046	0.319	0.750
LEQ × AQS^2^	−0.064	0.263	−0.046	−0.242	0.809
Child age	−0.065	0.131	−0.170	−0.498	0.618

Abbreviations: AQS, Attachment Q‐sort; AQS^2^, Square of Attachment Q‐sort Security Score; LEQ, Life Events Questionnaire.

**FIGURE 2:**
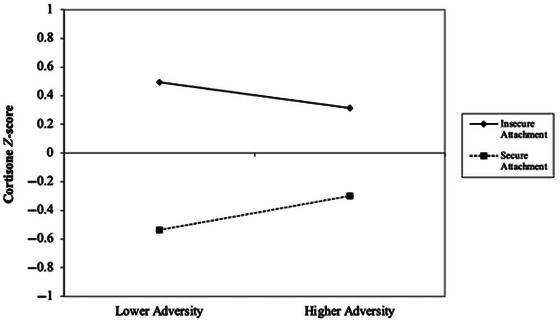
Two‐way linear effects estimated via regression analysis for adversity × Attachment Q‐sort on hair cortisone (all variables standardized, pooled multiple imputation data, *N* = 67).

For the cortisone analysis, attachment security was associated with lower cumulative cortisone (*p* = 0.001), suggesting a promotive effect (see Table 3). Although the AQS quadratic term and its interaction with adversity were not significant, the interaction term between the adversity score and the AQS was statistically significant (*p* = 0.049). The findings were similar following the addition of the child age variable. The statistically significant interaction term was explored by performing a graphing procedure (Figure 2), which allows one to visualize main and interactive effects simultaneously, along with a simple slopes analysis (Aiken et al. 1991; Brambor et al. 2006). The graphing procedure indicated that for children with higher attachment security scores, there was a positive association between adversity and cortisone. Simple slopes analysis, although not statistically significant, *t*(64) = 0.06, *p* = 0.953, revealed that the gradient of the slope was 0.296 for children with high attachment security scores. In contrast, for children with lower attachment security scores, the gradient of the slope was −0.228, although this was not statistically significant, *t*(64) = −0.09, *p* = 0.930.

**TABLE 3:** Regression results for hair cortisone (pooled, *N* = 67).

Variable	*B*	*SE*	*β*	*t*	*p*
Model 1					
Intercept	0.132	0.115		1.151	0.250
LEQ	−0.045	0.267	−0.147	−0.167	0.869
AQS	−0.561	0.173	−0.710	−3.233	0.001^**^
AQS^2^	0.170	0.162	0.285	1.055	0.292
LEQ × AQS	0.346	0.170	0.326	2.033	0.049^*^
LEQ × AQS^2^	−0.256	0.266	−0.203	−0.962	0.338
Model 2					
Intercept	0.135	0.116		1.167	0.244
LEQ	−0.053	0.267	−0.146	−0.199	0.843
AQS	−0.582	0.180	−0.685	−3.233	0.001^**^
AQS^2^	0.168	0.163	0.296	1.034	0.301
LEQ × AQS	0.349	0.171	0.319	2.041	0.048^*^
LEQ × AQS^2^	−0.256	0.267	−0.199	−0.960	0.339
Child age	0.057	0.128	−0.078	0.443	0.658

Abbreviations: AQS, Attachment Q‐sort; AQS^2^, Square of Attachment Q‐sort Security Score; LEQ = Life Events Questionnaire.

*p < 0.05; **p < 0.01.

In the analysis with the sum of standardized hair cortisol and cortisone as the outcome variable, attachment security was associated with lower combined hair cortisol/cortisone (*p* = 0.027), and it remained significant (*p* = 0.035) after the addition of child age (see Table 4). Neither of the interaction terms was statistically significant in either step.

**TABLE 4:** Regression results for the sum of standardized hair cortisol and cortisone (pooled, *N* = 67).

Variable	*B*	*SE*	*β*	*t*	*p*
Model 1					
Intercept	0.187	0.200		0.934	0.351
LEQ	−0.157	0.415	−0.167	−0.379	0.706
AQS	−0.678	0.307	−0.487	−2.207	0.027^*^
AQS^2^	0.094	0.281	0.129	0.336	0.737
LEQ × AQS	0.402	0.262	0.219	1.532	0.127
LEQ × AQS^2^	−0.321	0.438	−0.145	−0.733	0.464
Model 2					
Intercept	0.187	0.202		0.923	0.356
LEQ	−0.156	0.418	−0.166	−0.374	0.709
AQS	−0.675	0.320	−0.442	−2.106	0.035^*^
AQS^2^	0.095	0.283	0.149	0.335	0.738
LEQ × AQS	0.401	0.265	0.207	1.510	0.132
LEQ × AQS^2^	−0.320	0.442	−0.139	−0.724	0.469
Child age	−0.009	0.213	−0.140	−0.041	0.968

Abbreviations: AQS, Attachment Q‐sort; AQS^2^, Square of Attachment Q‐sort Security Score; LEQ = Life Events Questionnaire.

*p < 0.05.

Taken together, the most robust finding of these analyses was the significant association between attachment security and lower stress hormones.

## 4 Discussion

Children and families affected by parental incarceration often experience additional adversities; as such, it is necessary to balance assessments of vulnerabilities with examinations of resilience. The current study sought to examine the complex associations between incarceration, other childhood adversities, child–caregiver attachment relationships, and physiological stress. The results indicated that secure child–caregiver attachments were associated with lower cumulative stress hormones during their parents’ jail stay. These findings point to secure attachment relationships as a resilience factor for children with incarcerated parents, wherein stress processes are oftentimes thought of as a potential mechanism toward maladjustment.

Having a parent incarcerated is often stressful for children, and on average, it is associated with less optimal well‐being and the experience of additional ACEs (Poehlmann‐Tynan and Turney 2021). In our sample, the modal number of adversities that children experienced in a year's time was 4, with a mean of 5.16 (in addition to the ACE of parental incarceration). In Turney's (2018) study of a nationally representative sample, the average number of ACEs was 2 for children (age 0–17) with incarcerated parents, with higher numbers for children in the 0–6 age group, similar to our study. Murphey and Cooper (2015) discuss the potency of parental incarceration as an ACE for children younger than 6 years, finding that these young children with incarcerated parents have 1.6 more ACEs than children in that age range who have never experienced parental incarceration. Clearly, young children are particularly vulnerable to experiencing additional adversity in challenging situations such as parental incarceration, and the potential impact on their development is likely to be high because of the rapid growth and brain development that occur during early childhood (Smith and Pollak 2020).

In this study, we found that a child's secure attachment relationship to a caregiver was a possible resilience factor, as attachment security was associated with lower levels of combined cortisol/cortisone during the parent's incarceration. Foundational work has frequently called for the linkage between attachment and child stress (e.g., Kobak et al. 2015), to which this study provided the first evidence of *physiological* stress specific to a sample of children with incarcerated parents. Previous studies exploring attachment and perceived stress and coping have provided some preliminary evidence supporting this study's findings. For instance, in a sample of boys diagnosed with disruptive behavior disorders, Ooi and colleagues (2006) found that higher quality parent–child relationships as reported by parents were associated with lower levels of child‐reported social stress. Furthermore, Zimmer‐Gembeck and colleagues (2017) reviewed research examining the association between child–caregiver attachment relationships and their use of emotion regulation or coping strategies during toddlerhood through adolescence. The authors suggest that attachment security is associated with children's use of emotion regulation and coping strategies, though the reviewed studies evidenced mixed findings. Importantly, research has documented the influence of additional contextual factors on the link between parenting and children's physiological stress (e.g., parental vs. nonparental figure, age at time of adversity), necessitating additional work accounting for this complexity (Holochwost et al. 2020). More research is needed to explore the nuances of children's attachment relationships and physiological stress in diverse contexts of adversity, such as parental incarceration.

The LTA provides a valuable framework for understanding the relationship between attachment security and children's stress responses found in the current study, particularly in the context of adverse life experiences. According to LTA, secure attachment develops through repeated “learning events” where a caregiver effectively alleviates a child's distress, leading to reduced neuroendocrine stress markers, such as cortisol and cortisone. In this study, the significant linear and quadratic associations between attachment scores and cortisone levels reflect this process. Securely attached children, who likely experienced consistent, supportive responses from caregivers, exhibited lower overall cortisone levels as well as lower cortisol/cortisone levels, aligning with LTA's predictions. Conversely, the extreme cortisone levels observed in children with the most insecure attachments may reflect inconsistent or inadequate caregiver responses during critical learning events, underscoring the profound impact of attachment security on children's physiological stress regulation in the face of adversity.

There are multiple pathways through which secure attachments may help regulate a young child's stress reactivity. For example, having a safe, trusting adult available to children throughout the stressors that parental incarceration can bring may improve the child's ability to self‐regulate, have higher self‐esteem, seek out support, and share feelings in a way that those with less positive caregiver–child relationships are not able to. Recent meta‐analytic work by Pallini et al. (2018) supports these potential connections, as the authors document a modest positive association between attachment security (both measured continuously and categorically) and children's development of effortful control, an aspect of children's self‐regulation. In turn, children with higher attachment security scores, which are indicative of secure attachment relationships, may exhibit more regulated physiological stress responses, supporting more normative functioning and development. Hostinar and Gunnar (2013) discuss how experiences early in life become embedded within a child's stress regulation systems and shape their responses to future stressors. For example, Dagan et al. (2018) reported a moderating effect of adult attachment security on the link between telomere length (an indicator of cellular aging) and childhood adversity, where the number of adverse experiences predicted shorter telomere length only for individuals with insecure–dismissing attachments. Given these links, attachment interventions, such as the Attachment and Biobehavioral Catchup (Dozier and Bernard 2019), may be beneficial for families navigating ACE‐related stressors, including parental incarceration.

It is worth noting that the association between attachment and stress hormones was only present for the hair cortisone and the combined hair cortisol/cortisone variables, and not for hair cortisol on its own. There are a number of potential explanations for this finding. One reason to consider is that attachment‐mediated regulation of the HPA axis can occur at the central and/or local levels. There is a large body of literature demonstrating that secure attachments can mediate stress responsiveness of children via the regulation of the central HPA axis (e.g., Hostinar and Gunnar 2013; Kidd et al. 2011). In addition to central regulation, physiological stress regulation could also occur locally in hair, as 11beta‐hydroxysteroid dehydrogenase type 1, the enzyme that converts cortisone to cortisol, is present in hair follicles (Lee et al. 2017). Given that our study's measurement of physiological stress relied exclusively on hair samples, it may be that this regulation is being picked up more due to our methodology in a way that other samples (such as blood, saliva, or urine) may not. Second, because cortisol levels can fluctuate more in response to short‐term stressors, combining cortisol with cortisone may provide a more reliable and integrative marker of long‐term HPA axis activity; this approach has been used in prior research on children exposed to parental arrest, where the combined measure was well‐suited to capture chronic stress exposure (Muentner et al. 2021).

That said, there is evidence from validation work that hair cortisone is a marker of blood cortisol levels. For example, a study of radiolabeled cortisol administered systematically showed that it was incorporated into hair as both cortisol and cortisone (Kapoor et al. 2018). In addition, cortisone in saliva has been found to be a better marker of free blood cortisol levels, compared to cortisol in saliva (Perogamvros et al. 2010). Third, others have also shown that hair cortisone, instead of hair cortisol, is associated with diverse outcomes, including fatigue (Zhang et al. 2021), Parkinson's disease (van den Heuvel et al. 2020), and child maltreatment (Pittner et al. 2020), reinforcing the importance of the hair cortisone measure. Finally, it is important to note that this study used LC–MS/MS for hair glucocorticoid analysis, which is considered the “gold standard” assessment because of the measurement's specificity. Many studies measuring hair cortisol use immunoassay measures (e.g., Orta et al. 2018, Albar et al. 2013), which can have considerable cross‐reactivity with cortisone, and therefore scholars may inadvertently attribute findings to hair cortisol when cortisone may also have been measured. These points suggest that hair cortisone may have an important role as a biomarker of central HPA axis activity.

Resilience science is often critiqued as being complacent within and justifying the inequitable, systemic systems of oppression that create the need for resilience rather than challenging the sources of adversity (Masten 2014). While at times this is indeed the case, the work of dismantling systems takes enormous amounts of time (generations of time) during which children and families continue to be impacted by these adversities. Alongside social justice advocacy, decarceration efforts, and policy change, it is important to examine possible resilience processes already present in daily family life to begin applying existing interventions and develop new ones to support children with incarcerated parents and their families (Arditti and Johnson 2022). Indeed, Stern and colleagues (2022) call for the implementation of interventions that are informed by attachment theory to reduce systemic inequities in family support. Currently, few interventions exist that are tailored to young children affected by parental incarceration, and even fewer are available for caregivers (Poehlmann‐Tynan and Arditti 2018). Existing interventions, such as Circle of Security (The Circle of Security International 2019) and Strengthening Families Program (Kumpfer et al. 1989), have been piloted with children whose parents are involved in the criminal legal system or with caregivers (e.g., Cassidy et al. 2010; Miller et al. 2014), yielding positive preliminary outcomes. Additionally, mentoring programs appear to be the most popular in the community (Hagler et al. 2019), and parenting programs are growing within corrections facilities (Loper et al. 2019). More recently, interventions grounded in LTA (e.g., Video‐feedback Intervention to promote Positive Parenting and Sensitive Discipline [VIPP‐SD] and Middle Childhood Attachment‐based Family Therapy [MCAT]) have shown promise in fostering secure attachment and improving emotion regulation among children (Bosmans et al. 2022). Although no known studies have examined the use of LTA‐based interventions among children with incarcerated parents and their caregivers, this represents a promising direction for future research and clinical application, particularly given the relevance of attachment and stress processes in this population, as evidenced in the current study.

### 4.1 Limitations

Despite the contributions of this work, there are several limitations. First, the study's sample size is relatively small, and a larger group of children may elucidate more robust links between adverse events, attachment, and physiological stress. It is interesting to note that at the lowest levels of attachment, children in the present study had either very low or very high levels of cortisone, consistent with descriptions of hyper‐ and hypostress hormone reactions in children experiencing trauma. However, larger sample sizes are needed to further explore this.

Given the limited statistical power that comes with a smaller sample, there may also be confounding variables omitted from the models, such as access to other protective factors (e.g., social support, attachments to extended family members) or exposure to other adverse or traumatic events (e.g., witnessing parents’ arrest, separations from primary caregiver). For example, in the current study, the exact length of time that the child had lived with their current caregiver was unknown, and some caregivers reported a few separations from the child since the child had been living with them. Both children's attachment security to their caregivers and children's physiological stress may be influenced by the stability and length of the caregiving arrangement (e.g., Fisher et al. 2011; Pasalich et al. 2016), meaning that this may be an important confounding variable missing in the current analyses. Future work should assess whether the length of time the child has lived with their current caregiver, and any separations experienced from the caregiver, influences attachment security and physiological stress.

Additionally, although the current study examined low and high adversity among a sample of children with incarcerated parents, no comparison group existed of children who did not experience parental incarceration, thus limiting the findings to children who have experienced the incarceration of a parent. Further, the temporal ordering of events is challenging to deduce given the cross‐sectional nature of the study. Future work could employ a longer‐term follow‐up of children. Longitudinal research would help to understand physiological stress in children with incarcerated parents, including identification of protective factors that can facilitate resilience processes and increase our understanding about how physiological stress is associated with children's longer‐term outcomes.

Additional limitations come with measuring stress hormones through hair, such as variability in growth rates across individuals and the impact of medication use or seasonal variation. In future work, it is important to incorporate a systemic measure of cortisol and cortisone from blood or saliva, in addition to hair cortisol and cortisone, to determine if the findings using other measures mirror the results found using hair. Additionally, other measurement approaches may be more applicable for other research questions. For instance, samples of saliva that measure more acute stress levels could be used to assess the protective role of caregiver–child attachments before and after visiting a parent in jail or prison. Importantly, the findings may not be generalizable to all those with incarcerated parents, given the study's focus on young children, jail incarceration, and data collection in locales with disparities in incarceration rates above the national average.

## 5 Conclusion

This study provides the first evidence of links between ACEs, child–caregiver attachment, and physiological stress in a sample of young children with jailed parents. Given the significant concentration of adversity among young children with incarcerated parents (Turney 2018; Murphey and Cooper 2015), positive child–caregiver relationships were posited to be associated with more regulated physiological stress responses. Findings yielded support for this conclusion, such that those young children with higher attachment security scores had lower levels of cortisone and the combined cortisol/cortisone variable, despite negative life events that they may have experienced. These findings call for investigations of prevention and intervention programs that focus on the family system to strengthen relationships between caregivers and children to mitigate the long‐term consequences that chronic stress can have on children (and particularly those with less secure attachments). At a time when the United States is grappling with the collateral consequences of mass incarceration, the results call for more attention to be placed on the well‐being of children whose parents are in jail and the family processes that buffer these consequences. Indeed, a dedicated social justice–oriented approach to serving impacted children has the potential to strengthen family relationships and promote more optimal child outcomes.

## Conflicts of Interest

The authors declare no conflicts of interest.

## Data Availability

The data that support the findings of this study are available on request from the corresponding author. The data are not publicly available due to privacy or ethical restrictions.
